# Metabolic effects of quail eggs in diabetes-induced rats: comparison with chicken eggs

**DOI:** 10.3402/fnr.v60.32530

**Published:** 2016-10-06

**Authors:** Eric Lontchi-Yimagou, Agatha Tanya, Carine Tchankou, Judith Ngondi, Julius Oben

**Affiliations:** 1 Laboratory for Molecular Medicine and Metabolism, Biotechnology Center, University of Yaoundé I, Yaoundé, Cameroon; 2 Department of Public Health, Faculty of Medicine and Biomedical Sciences, University of Yaoundé I, Yaoundé, Cameroon; 3 Laboratory of Nutrition and Nutritional Biochemistry, Faculty of Sciences, University of Yaoundé I, Yaoundé, Cameroon

**Keywords:** diabetes, hyperlipidemia, oxidative stress, quail eggs, chicken eggs

## Abstract

**Background:**

Quail eggs as a food item have recently been introduced into the diet of some Cameroonians. These eggs are being sold in local markets, but with many unfounded health claims. One claim is that quail eggs can reduce blood glucose levels in diabetics. It was therefore necessary to evaluate the effect of consuming quail eggs on blood glucose levels, lipid profiles, and oxidative stress parameters in diabetes-induced rats.

**Methods:**

Twenty Wistar rats weighing, on average, 250 g were divided into four groups of five rats each. Group 1 consisted of rats with normal blood glucose, and the other three groups (2, 3, and 4) consisted of diabetes-induced rats achieved by intravenous injection of streptozotocin. During 16 days, rats in groups 1 and 2 received distilled water; and rats in groups 3 and 4 received quail and chicken eggs, respectively, with gastroesophageal probe at a dose of 1 mL/200 g body weight. Fasting blood glucose levels were determined in all the groups on the 1st, 7th, 14th, and 17th days after induction of diabetes. On the 17th day, the fasting rats were sacrificed, and blood and liver samples were collected for biochemical analyses.

**Results:**

In 17 days, the consumption of quail and chicken eggs had no effect on blood glucose levels of diabetic rats. Total cholesterol levels were higher in groups 3 (75.59 mg/dL) and 4 (59.41 mg/dL) compared to group 2 (55.67 mg/dl), although these differences were not significant (all *p*>0.05). Triglyceride levels were significantly higher (*p*<0.05) in groups 3 (106.52 mg/dL) and 4 (109.65 mg/dL) compared to group 2 (65.82 mg/dL). Quail eggs had no effect on oxidative stress parameters (malondialdehyde, hydroperoxides, and catalase).

**Conclusions:**

The consumption of quail eggs by diabetic rats at the tested dose had no effect on blood glucose level and oxidative stress parameters and may have a negative effect on lipid profile.

The prevalence of type 2 diabetes mellitus (T2DM) has increased worldwide (1, 2), including in Africa, where the greatest proportional increase (90%) in the number of diabetic cases is projected to occur by 2030 (3, 4). Sedentary lifestyles, obesity, and unhealthy dietary behaviors are the most common risk factors of T2DM (5). T2DM leads to serious complications such as nervous system disorders, kidney diseases, and eye pathologies; thus, its prevention and treatment should be considered a priority (6). Diet has been used in the prevention and management of T2DM. Strategies used to manage obesity include the use of high-fiber, low-carbohydrate, and low-fat diets (7, 8). Conventional dietary and behavioral treatments have failed in long-term management.

The Japanese quail (Galliformes: Phasianidae) has existed since ancient times. Consumption of Japanese quail eggs has been claimed to improve metabolism; prevent stress; and help in the treatment of obesity, asthma, and various allergies (9). These eggs are rich sources of antioxidants, minerals, vitamins, and other nutrients (10). Reportedly, quail eggs strengthen the immune system, promote memory health, increase brain activity, and stabilize the nervous system (11). They apparently help relieve anemia by increasing the level of hemoglobin in the body while removing toxins and heavy metals. After the consumption of quail eggs, Anca et al. (12) stated that ‘in Brasil, people claim to be relieved of many physiological disorders such as anemia, tuberculosis, ulcers, hypertension, diabetes, arteriosclerosis, asthma and many others’. Aba and Onah (13) showed that alloxanized rats that were administered a quail egg solution demonstrated hypolipidemia and ameliorated lesions in the pancreas. Quail eggs have recently been introduced in Cameroon. They are sold in local markets and some supermarkets at exorbitant prices and with many nutritional claims. They are appreciated for their nutritional value and functional properties. However, without any real justification, people believe that consuming these eggs can relieve diabetes, hypertension, asthma, sinusitis, cardiovascular diseases, and many other conditions. The public health consequences of such allegations may be serious; therefore, this work was undertaken to address this information gap by evaluating of the effects of quail egg consumption on the blood glucose level, lipid profile, and oxidative stress parameters in diabetes-induced rats.

## Methods

### Animal management and ethical considerations

Adult male Wistar albino rats 12 weeks of age and weighing, on average, 250 g were used in this study. Rats (*n*=20) were purchased from the animal house of the Faculty of Medicine and Biomedical Sciences, University of Yaoundé I, Cameroon. The animals were maintained at 25°C in a room with a 12/12-h light/dark cycle and had free access to water and food. The study was approved by the institutional animal ethical committee.

### Quail and chicken eggs

To enable a comparative study on different groups of rats, we used both quail and chicken eggs. Both types of eggs were purchased from the same breeder in the Biyem-Assi neighborhood (Yaoundé-Cameroon) to avoid variations related to food quality that could modify the composition of eggs (14).

### Egg administration

Every morning, quail and chicken eggs were administered to each rat with a gastroesophageal probe at a dose of 1 mL/200 g body weight. This dose reflected the dose used by most Cameroonians for their treatment. The egg was broken, introduced into a beaker, and then homogenized using a glass rod. The volume of eggs that was given to the rats was drawn with a graduated syringe into the gastroesophageal probe for administration to the rats.

### Evaluation of nutritional effects of quail and chicken eggs on **blood glucose** of diabetes-induced rats

#### Induction of diabetes in rats

A single intraperitoneal dose of 55 mg/kg streptozotocin (STZ; Sigma-Aldrich, Germany) was administered to induce diabetes (15). After 72 h, rats with marked hyperglycemia (fasting blood glucose of 250 mg/dL) were considered diabetics. The animals were divided into four groups of five rats each. The four groups were treated as follows:

**Table:** 

Group	Description
1	Normal/untreated rats (negative control) receiving distilled water at the dose of 1 mL/200 g BW.
2	Diabetics/untreated rats (positive control) receiving distilled water at the dose of 1 mL/200 g BW.
3	Diabetics/treated rats receiving quail eggs at the dose of 1 mL/200 g BW.
4	Diabetics/treated rats receiving chicken eggs at the dose of 1 mL/200 g BW.

#### Blood collection

The effects of quail and chicken eggs on blood glucose, lipid profile, and oxidative stress parameters of the four groups of rats were determined. Group 1 consisted of rats with normal blood glucose and the other three groups (2–4) consisted of diabetes-induced rats by intravenous injection of STZ. For 16 days, groups 1 and 2 received distilled water; groups 3 and 4 received quail and chicken eggs, respectively, from a gastroesophageal probe at a dose of 1 mL/200 g BW. The rats were on these specific treatments for 16 days and had free access to water and were on the same diet. Fasting blood glucose levels were determined in all the groups on the 1st, 7th, 14th, and 17th days after induction of diabetes. On the 17th day, the fasting rats were sacrificed by jugular venesection after light chloroform anesthesia.

For each rat, blood was collected into ethylenediaminetetraacetic acid tubes and allowed to clot at room temperature. Thereafter, plasma was separated from the clot by centrifuging at 1,126*g* for 15 min. The plasma was collected in clean bottles and stored at −20°C until required. After the animals were dissected, organs were put on ice and processed as rapidly as possible.

- *Preparation of liver homogenates*: Approximately 10% (w/v) tissue homogenates were prepared in 10% phosphate-buffered saline (pH 6.4) by using a microhomogenizer (Physcotron, Niti-on Inc., Chiba, Japan). The supernatant was obtained from the homogenate after centrifugation at 3,000 rpm for 15 min. The sample was then stored at −20°C until used for the determination of malondialdehyde (MDA) concentration, hydroperoxides concentration, and the activities of catalase.
- *Preparation of hemolysates of erythrocytes*: After centrifuging total blood, 100 µL of pellets was pipetted into another tube and then washed twice with 2 mL of 0.9% NaCl and centrifuged at 1,126*g* for 10 min at room temperature. Hemolysates were obtained by adding 2 mL of distilled water and was stored at −20°C. This hemolysate was used for protein assays and determination of catalase activity.

### Biochemical analyses

Plasma was prepared from blood samples collected from rats on day 17 in the morning after an overnight fast of 12 h. Plasma glucose was determined using a GlucoPlus^™^ glucometer. Total cholesterol and triglycerides were measured enzymatically using standard enzymatic kits (Sigma Diagnostics). Oxidative stress markers such as MDA level, hydroxyl (OH) radical level, and activity of catalase were also measured.

### Evaluation of effect of quail and chicken eggs on lipid profile of diabetes-induced rats

Plasma total cholesterol and triglycerides were estimated enzymatically according to Watson (16) and Fossati and Prencipe (17), respectively, by using commercial kits from Ran-dox, Ltd., Co. (UK).

### Evaluation of antioxidant properties of quail eggs on diabetes-induced rats

- Total protein content was evaluated in all collected samples (hemolysate of erythrocytes, plasma and homogenates of liver) using the ‘total protein colorimetric test Biuret’ kit commercialized by Cypress Diagnostics.
- MDA level was estimated spectrophotometrically by thiobarbituric acid according to methods described by Wilbur et al. (18). Thiobarbituric acid reacts with plasma MDA that is produced by hydrolysis of lipid hydroperoxides to form a pink-red colored complex with high absorbance at 532 nm. The results are expressed as micromoles per liter. This complex is usually quantified against MDA standards that are generated from 1,1,3,3-tetraethoxypropane under the same reaction conditions.
- OH radical level was measured in blood plasma and homogenates of liver according to the protocol described by Jiang et al. (19). This method is based on the principle that in acid medium, the peroxide ion oxidizes Fe^2+^ into Fe^3+^ that then reacts with xylenol orange to form a complex that absorbs at 560 nm.
- Catalase activity in hemolysates and liver homogenates was assayed as previously described by Sinha (20). An aliquot of hydrogen peroxide (0.8 mL) was dispensed into an Eppendorf tube. Phosphate buffer (1.0 mL) and (0.002%, v/v) diluted homogenate/hemolysate (125 µL) were added. The reaction mixture (0.5 mL) was dispensed into 5% dichromate reagent (1.0 mL) and vigorously shaken. The mixture was heated in a Clifton water bath for 10 min and allowed to cool. The absorbance at 570 nm was taken using a spectrophotometer (BioMate 3S UV-Visible, Thermo Scientific, Wohlen, Switzerland). The absorbance obtained was extrapolated from the following standard curve: *y*=0.0028*x*+0.0132. The catalase activity was thereafter expressed as units per minute per milligram of protein: catalase (unit/mg protein)=(Abs/min×30,000 units)/(40 cm/M×mg protein)×df, where df is dilution factor and Abs is absorbance.

### Statistical analysis

Statistical analysis of the results was performed using SPSS 10.1 for Windows (SPSS, Chicago, IL, USA). The results are presented as mean±standard deviation. The comparison was done using one-way analysis of variance followed by Duncan's multiple range post hoc test. A value of *p*<0.05 was considered as significant.

## Results

### Evaluation of nutritional effects of quail and chicken eggs on blood glucose of diabetes-induced rats

**Fig. 1:**
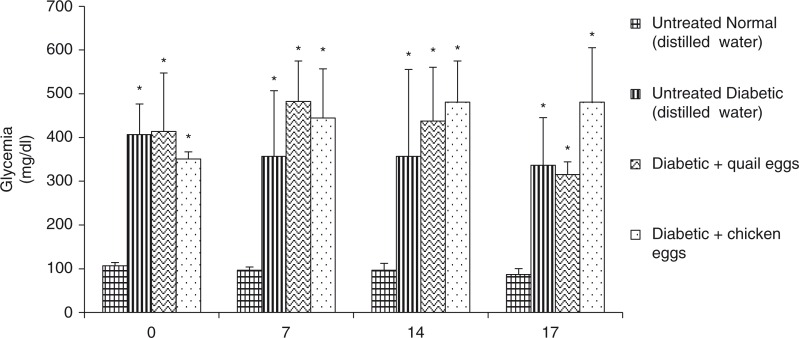
Nutritional effect of quail and chicken eggs at dose of 1 mL/200 g BW for 16 days on blood glucose in diabetes-induced rats. Data are means+standard deviation. *p<0.05, significantly different from untreated normal control (distilled water 1 mL/200 g BW).

The results of nutritional effect of quail and chicken eggs on blood glucose in diabetic rats are presented in Fig. 1. The administration of quail and chicken eggs for 16 days in diabetic rats had no effect on blood glucose. After 7, 14, and 16 days of treatment, there was no significant differences in blood glucose between the positive control diabetic rats receiving distillated water and diabetic rats treated with quail and chicken eggs (*p*>0.05).

### Evaluation of effect of quail and chicken eggs on lipid profile of diabetes-induced rats

**Fig. 2:**
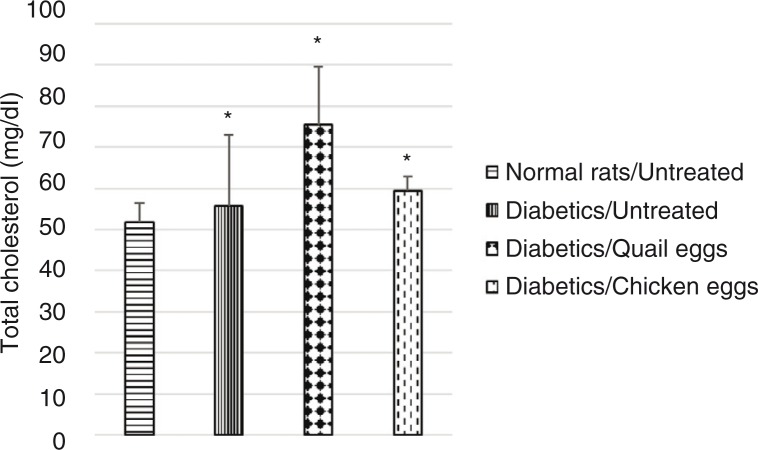
Nutritional effect of quail and chicken eggs at dose of 1 mL/200 g BW for 16 days on total cholesterol in diabetes-induced rats. Data are means+standard deviation. *p<0.05, significantly different from untreated normal control (distilled water 1 mL/200 g BW).

**Fig. 3:**
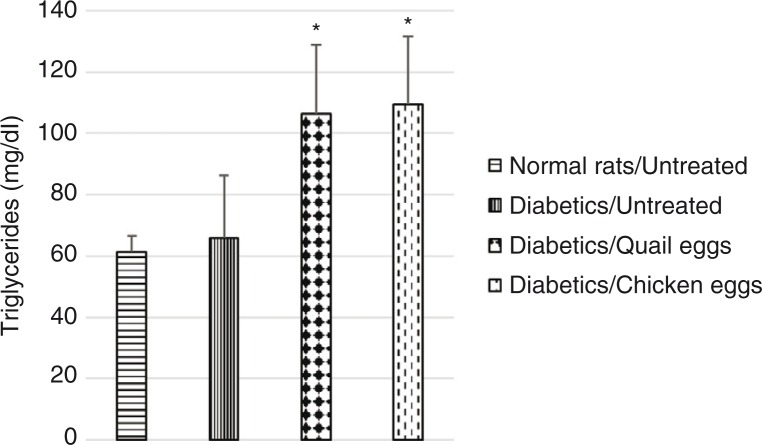
Nutritional effect of quail and chicken eggs at dose of 1 mL/200 g BW for 16 days on triglycerides in diabetes-induced rats. Data are means+standard deviation. *p<0.05, significantly different from untreated normal control (distilled water 1 mL/200 g BW) and untreated diabetic (distilled water 1 mL/200 g BW) rats.

Plasma total cholesterol was significantly elevated in untreated diabetic rats compared to normal untreated rats. Total cholesterol level was higher in diabetic rats receiving quail eggs (75.59 mg/dL) and those receiving chicken eggs (59.41 mg/dL) compared to non-treated diabetic rats (55.67 mg/dL), although this difference was not significant (*p*>0.05) (Fig. 2). Also, triglyceride levels were significantly higher (*p*<0.05) in diabetic rats receiving quail eggs (106.52 mg/dL) and chicken eggs (109.65 mg/dL) compared to diabetic untreated rats receiving distilled water (65.82 mg/dL) (Fig. 3).

### Evaluation of effect of quail eggs on OH radical and MDA levels in diabetes-induced rats

Plasma and liver homogenates OH radical and MDA levels were significantly elevated in untreated diabetic rats compared to normal untreated rats (*p*<0.05). During the 16 days, no difference was observed in these parameters in untreated diabetic rats compared to diabetic rats treated with quail and chicken eggs (*p*>0.05) (Tables 1 and 2).

**Table 1:** Effect of quail and chicken eggs on hydroperoxides in diabetes-induced rats

	Oxidative stress parameter (hydroperoxides)	
	
Group of rats	Plasma	Liver homogenate
Untreated normal (distilled water 1 mL/200 g BW)	0.3±0.01	0.5±0.1
Untreated diabetic (distilled water 1 mL/200 g BW)	0.6±0.1*	0.8±0.1*
Diabetic+quail eggs (1 mL/200 g BW)	0.7±0.1*	0.9±0.3*
Diabetic+chicken eggs (1 mL/200 g BW)	0.5±0.04*	0.9±0.1*

Data are means±standard deviation. BW=body weight.

* *p*<0.05, significantly different from untreated normal control (distilled water 1 mL/200 g BW).

**Table 2:** Effect of quail and chicken eggs on the malondialdehydes in diabetes induced rats

	MDA (µmol/L)	
	
Group of rats	Plasma	Liver homogenate
Untreated normal (distilled water 1 mL/200 g BW)	5.9±0.6	3.5±0.2
Untreated diabetic (distilled water 1 mL/200 g BW)	10.8±1.3*	10.8±0.3*
Diabetic+quail eggs (1 mL/200 g BW)	9.3±1.4*	8.3±1.3*
Diabetic+chicken eggs (1 mL/200 g BW)	7.1±1.2*	7.4±1.7*

Data are means±standard deviation. BW=body weight; MDA=malondialdehyde.

* *p*<0.05, significantly different from untreated normal control (distilled water 1 mL/200 g BW).

### Evaluation of effect of quail eggs on catalase activity in diabetes-induced rats

**Fig. 4:**
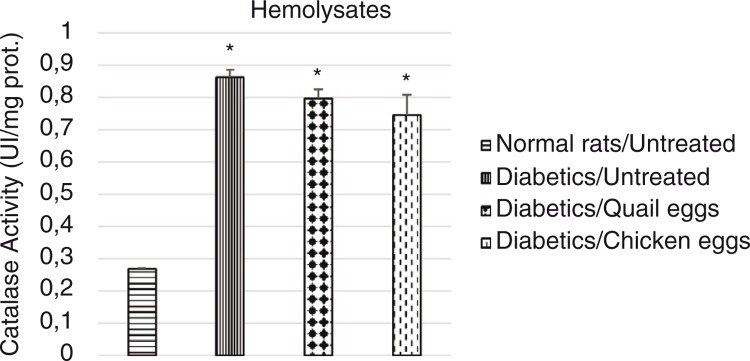
Effect of quail and chicken eggs on the catalase activity (hemolysates) in diabetes-induced rats. Data are means+standard deviation. *p<0.05, significantly different from untreated normal control (distilled water 1 mL/200 g BW).

**Fig. 5:**
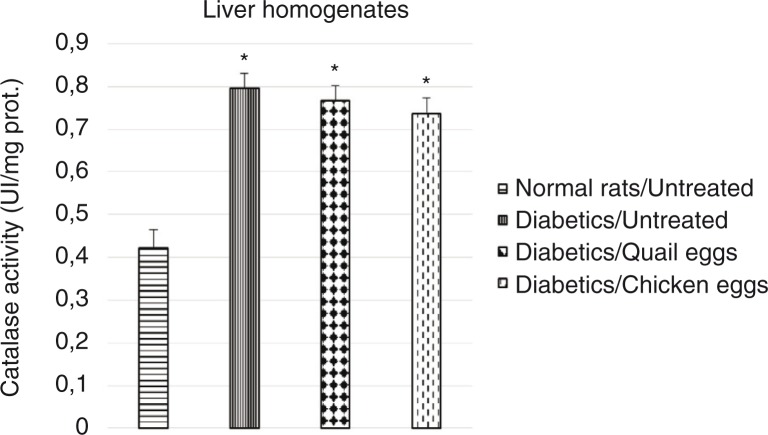
Effect of quail and chicken eggs on the catalase activity (liver homogenates) in diabetes-induced rats. Data are means+standard deviation. *p<0.05, significantly different from untreated normal control (distilled water 1 mL/200 g BW).

The effect of the consumption of quail eggs on the activity of catalase on hemolysates and liver homogenates in diabetes**-**induced rats is shown in Figs. 4 and 5, respectively. The catalase activity in hemolysates and liver homogenates was significantly elevated in untreated diabetic rats compared to normal untreated rats (*p*<0.05). Higher catalase activity was observed in hemolysates and liver homogenates in the untreated diabetic rats compared to the treated diabetic rats, although the difference was not significant (*p*>0.05).

## Discussion

Diabetes induced with STZ lead to a significant elevation of blood glucose. This result is consistent with that of Bibak et al. (15). We observed an increase of total cholesterol and triglyceride levels and markers of lipid peroxidation (MDA and hydroperoxides) and catalase in STZ-induced diabetic rats. Diabetes induced with STZ in rats is a good disease model that can be used to test substances that are supposed to have hypoglycemic, hypolipidemic, and antioxidant activity *in vivo*. STZ is an antibiotic that produces pancreatic islet β-cell destruction and is widely used experimentally to produce a model of type 1 diabetes mellitus. These animals are used for assessing the pathological consequences of diabetes and for screening potential therapies for the treatment of this condition (21, 22).

The consumption of quail eggs had no significant effect on blood glucose levels in treated rats compared to untreated diabetic rats. This could be an indication that quail eggs had no substance that can affect blood glucose. These results are contrary to those reported by Anca et al. (12) and the beliefs of Cameroonians who use quail eggs for the treatment of diabetes. It is possible that for some people with diabetes who consume quail eggs and are also taking oral hypoglycemic agents or insulin, the components of quail eggs could have a synergistic effect with the bioactive compounds contained in drug formulations. For those who consume quail eggs without any medication, the improvement in their disease may simply be a psychological effect. Therefore, it will be interesting to also carry out this study directly in humans. However, at this stage of the research, one should be wary of the exclusive use of quail eggs in the management of diabetes.

An increase was observed in the levels of lipid profile parameters in rats rendered diabetic with STZ. In 16 days, treatment of diabetic rats with quail and chicken eggs resulted in an increase of the concentration of total cholesterol and triglyceride levels compared to those of untreated diabetic rats, although this difference was not significant regarding the level of total cholesterol. This trend of increased total cholesterol levels in rats treated with quail and chicken eggs compared to the untreated diabetic rats could be due to the high level of cholesterol in the eggs. Bragagnolo and Rodriguez-Amaya (23) showed that the concentration of cholesterol in quail eggs is similar to that found in chicken eggs: 12.1 mg of cholesterol per gram of yolk in quail eggs compared to 12.0 mg in chicken eggs. These results are contrary to the results of Aba et al. (13) who observed a reduction in the serum levels of total cholesterol, low-density lipoprotein cholesterol, and very-low-density lipoprotein cholesterol and an increase in high-density lipoprotein cholesterol in a concentration-dependent manner compared to the untreated diabetic group. The American Heart Association recommends a daily cholesterol intake of <300 mg for healthy individuals and <200 mg for at-risk individuals. Eggs are one of the main sources of dietary cholesterol, with a large egg containing about 200 mg of cholesterol (24). Cameroonians consume for their treatment, on average, 12–14 quail eggs per day. It would be advisable for them to reduce their daily consumption of quail eggs.

Diabetes induced with STZ is a good disease model that can be used to test substances that are supposed to contain antioxidant activity *in vivo*. Increased oxidative stress is widely accepted as a major factor in the development and progression of diabetes mellitus (25). The obtained experimental diabetes in laboratory animals such as rats is a good model to study oxidative stress due to chronic hyperglycemia, which thereby depresses the activity of the antioxidant defense system and induces the generation of free radicals *de novo*. The free radicals thus formed react with all biological substances. The reactions with these constituents of the cell membrane lead to lipid peroxidation (26). The liver contains a high content of antioxidant enzymes such as catalase and superoxide dismutase that can trap these free radicals. Compounds with antioxidant properties should be able to reduce the concentration of free radicals. The lipid peroxidation in diabetes leads to increased plasma and hepatic levels of MDA and hydroperoxides. This study did show an effect of the consumption of quail and chicken eggs on markers of oxidative stress. Notably, the levels of MDA and hydroperoxides in the plasma and liver homogenates increased in untreated diabetic rats, showing an increased generation of free radicals compared to those of normal rats. The increase in MDA and hydroperoxide levels in untreated diabetic rats might have resulted from the decreased activity of antioxidant enzymes. A decrease in the activity of catalase was also observed. There was no significant difference in oxidative stress markers between the groups treated with quail and chicken eggs with respect to untreated groups. These results suggest that quail eggs might not contain any substance with antioxidant properties.

## Conclusions

The present investigation has revealed that the consumption of quail eggs by diabetic rats has no direct effect on blood glucose, but oxidative stress parameters may have a negative effect on the lipid profile. Depending on the parameters that have been studied in diabetic rats, quail eggs may be considered to have the same properties as chicken eggs. Quail eggs are richer in cholesterol compared to chicken eggs and require caution in their consumption.
